# A New Non-Surgical Treatment for Inflammatory Exudates and Their Residua in the Female Pelvis

**Published:** 1901-08

**Authors:** Hugo Ehrenfest

**Affiliations:** St. Louis, Mo.; Consulting Gynecologist to the City Hospital


### A NEW NON-SURGICAL TREATMENT FOR INFLAMMATORY EXUDATES AND THEIR RESIDUA IN THE FEMALE PELVIS.*

By HUGO EHRENFEST, M.D., St. Louis, Mo.
Consulting Gynecologist to the City Hospital.

During the last few years much stress has been laid on the attempt to treat acute and chronic inflammatory conditions of the female pelvis and their residua by methods of a less radical nature. While panhysterectomy continues to be the only resource in certain cases, it was proven on the other hand, that some conservative measures give equally as good and, in some cases, even better results. These conservative methods are both operative and non-operative. The operative methods attempt to remove only the diseased portion of the genital organs, thereby preserving (should such be possible) the function of menstruation and the possibility of pregnancy. These conservative, operative procedures are mainly

*Read before the Medical Society of City Hospital Alumni, March 7, 1901.

recommended and employed by American gynecologists. In some contradiction to them, German authors are attaching more value to the conservative, *non-operative* methods. It seems to me that these methods do not get the appreciation in America which they merit.

A review on the new book of Professor Macnaughton-Jones, of London, in the *New York Medical Record*, December 22, 1900, p. 990, contains the followiug sentence: "The last chapter, which includes the subject of 'Massage,' would better have been omitted, since mechanical treatment is to-day hardly looked upon by us as a justifiable, let alone a legitimate aid to the cure of disease peculiar to women."

According to my own experience and the results given by a great many trustworthy authors, this view is not justified and in many respects dangerous. The value of some conservative, non-surgical procedures is at present, without doubt, established. To this class belong—gynecological massage, hot-water douches, the treatment with the thermophor, etc. With this paper I wish to call your attention to a new procedure, comparatively young and unknown. Being well acquainted with all the above-mentioned methods, I think this new one offers some remarkable advantages. Very favorable results which I have experienced by employing it justify me in recommending the same. In brief, allow me to give you the origin of this new procedure.

Professor W. A. Freund, of Strasburg, inaugurated a method of treatment of pelvic exudates by "pressure-weight," and presented a paper on this subject at the "Naturforscher-Versammlung," at Braunschweig, in 1897. The pressure is produced by placing a bag filled with bird-shot on the abdomen, and at the same time inserting into the vagina a rubber condom also filled with shot.

Funke* reported a series of cases from Freund's clinic, in which excellent results followed this treatment, and gave in this report the indications for the use of this method. Before the above was published, Pincus† used and recommended a similar treatment, to which he gave the name of "pressure-posture." His method con-

*Hegar's Beitraege, Bd. 1, p. 264.
†Zeitschrift f. Geb. und Gyn, Bd., 39, p. 13.

sists of placing the patient in the Trendelenburg position with a bag of shot on the abdomen, and nothing in the vagina save a colpeurynter filled with air, its object being to support the uterus.*

The many successful cases following the use of these methods induced my former chief, Professor Schauta of Vienna, to try them in his clinic. Schauta did not limit himself to the use of either method, but utilized a combination of both, which consisted in placing a shot bag on the abdomen and at the same time introducing a colpeurynter filled with metallic mercury into the vagina, the patient being placed in the Trendelenburg position.

The most satisfactory results with this method were published by Halban.† Later, Funke‡ advised some changes in the technique, which I have adopted, and I find them of great practical value.

I will now give you a short account of the rationale, the indications and the technique of this procedure:

The shot bag placed on the lower part of the abdomen compresses the pelvic exudations, while counter-pressure is produced by the vaginal colpeurynter filled with mercury; the latter exerts the same influence in the reversed direction, with the addition, that the uterus is forced out from the lower pelvis, thereby stretching all adhesions and bands, if any exist. For this particular purpose the Trendelenburg posture is the most effective. In this posture the mercurial weight does not, as in the horizontal dorsal position, press against the sacrum, but it exerts its influence in the direction of the pelvic axis and tends to press the uterus in the same direction.

In the case of a retroflected uterus, pregnant or non-pregnant, the colpeurynter placed into the posterior fornix will push the uterus directly out of the cul-de-sac and cause it to assume an anteverted position, the patient being in the elevated dorsal posture. By changing the position of the patient to either side we can produce the pressure in any direction desired. Such treatment further assists the absorption of exudates by improving the pelvic circulation.

---

*This method is spoken of by John C. Clark, *Am. Gynecol. and Obstet. Journ.*, April, 1900.

†Monatschrift f. Geb. und Gyn., February, 1899.

‡Centralblatt f. Gyn., No. 3, 1900.

The *indications* for the use of this method are found in cases of chronic inflammatory conditions of the pelvic organs or their results, and in malpositions of the uterus.

Naturally we may expect the most favorable results in lesions situated in the lowest part of the pelvis, for instance, cicatrices following cervico-vaginal lacerations, hard exudates as the result of chronic parametritis, deep-seated adhesions and bands after perimetritis, as well as shortening of the sacrouterine ligaments. In adhesive bands of the fundus uteri the effect is not so manifest, though in such cases we often succeed in materially relieving the subjective symptoms due to the stretching of these bands. In the treatment of retroversion of the uterus, where the fixations are low down in the pelvis, the results of this method are surprisingly good. In cases of incarceration of the pregnant uterus, even a single introduction of the vaginal colpeurynter (filled with mercury) was followed by the correction of this abnormal condition (as reported by Halban and Funke).

The principal contraindications of the use of this method are all *acute* inflammatory conditions of the pelvic contents. In some cases of subacute conditions we may use this treatment, but only with the greatest caution, in order to avoid a recurrence of acute symptoms, particularly in cases of pyosalpinx. In such cases, when the tubes and their exudates are located in the cul-de-sac, we often get good results, if the proper precautions are observed. Care must be taken that in these cases the weight of the mercury in the vaginal colpeurynter is between 200 and 400 grams, and the treatment must be immediately stopped as soon as the patient complains of severe pain. Should the patient have an elevation of temperature, this method must not be repeated.

The general *technique* of this method is as follows:

Two vaginal colpeurynters are connected by means of a stopcock made of hard rubber, so that the whole length of the apparatus is about 60 cm. Before connecting this apparatus, one of the colpeurynters is filled with 1,000 grains of metallic mercury, while from the other the air is evacuated by compression of the bulb.

The patient is placed in a comfortable recumbent position on a bed or couch, the foot end of which is elevated about 50 to 60 cm.

The empty colpeurynter is folded, introduced into the vagina and placed against the part desired. It is retained in this position by means of two fingers, while the filled colpeurynter is elevated with the other hand, allowing the mercury to flow downward. In the beginning of the course of this treatment it is advisable not to use more than between 250 to 500 grams, and only after the patient has become accustomed to this treatment, we may increase the amount until the upper colpeurynter is completely emptied. The closed valve then prevents the return flow of the mercury. A flat linen bag, containing 1,500 to 2,000 grams of shot is placed on the lower abdomen.

The patient usually remains in the dorsal position, but should it be necessary to turn the patient on either side, the abdominal bag can be kept in position by means of a bandage. To remove the filled colpeurynter from the vagina, the patient is allowed to sit up, and by opening the valve and lowering the empty colpeurynter, the metal flows out readily.

The apparatus should be cleaned and kept in an antiseptic solution.

At the beginning of the treatment the procedure must not consume more than fifteen minutes; yet, I have found that patients become speedily accustomed to it, and it is by no means uncommon that patients remain in the Trendelenburg position with the pressure of 1,000 grams in the vagina for four or five hours with very little inconvenience. Usually I leave the colpeurynter in place for one or two hours every second day. As mentioned before, the treatment must be interrupted should the patient complain of pain, and in a few days it may be attempted again, but in the event of elevation of temperature this method must be given up, as this condition contraindicates its use.

The pressure treatment is a form of forced massage, but it does not exclude manual massage according to Thure Brandt's method.

We can treat more successfully the adhesions which are situated higher up in the pelvis by means of manual massage, after we have removed the exudates and adhesions below by pressure weight. My experience, as mentioned before, justifies me to strongly recommend this treatment. If applied according to the directions suggested in this paper, it will be followed by good, sometimes even by surprising results.

3301 Lucas Avenue.

